# Exploration of shared TF-miRNA‒mRNA and mRNA-RBP-pseudogene networks in type 2 diabetes mellitus and breast cancer

**DOI:** 10.3389/fimmu.2022.915017

**Published:** 2022-09-05

**Authors:** Wu Tong, Gu Wenze, Hong Libing, Cao Yuchen, Zhao Hejia, Guo Xi, Yang Xiongyi, Yi Guoguo, Fu Min

**Affiliations:** ^1^ The First Clinical School, Southern Medical University, Guangzhou, China; ^2^ The Second Clinical School, Southern Medical University, Guangzhou, China; ^3^ Department of Ophthalmology, The Sixth Affiliated Hospital of Sun-Yat-Sen University Guangzhou, Guangdong, China; ^4^ Department of Ophthalmology, Zhujiang Hospital of Southern Medical University, Guangzhou, Guangdong, China

**Keywords:** rT2DM, BC, miRNA-TF-mRNA network, RNA methylation, immune microenvironment, ceRNA

## Abstract

Type 2 diabetes mellitus (T2DM) has been confirmed to be closely associated with breast cancer (BC). However, the shared mechanisms between these diseases remain unclear. By comparing different datasets, we identified shared differentially expressed (DE) RNAs in T2DM and BC, including 427 mRNAs and 6 miRNAs from the GEO(Gene Expression Omnibus) database. We used databases to predict interactions to construct two critical networks. The transcription factor (TF)-miRNA‒mRNA network contained 236 TFs, while the RNA binding protein (RBP)-pseudogene-mRNA network showed that the pseudogene S-phase kinase associated protein 1 pseudogene 1 (SKP1P1) might play a key role in regulating gene expression. The shared mRNAs between T2DM and BC were enriched in cytochrome (CYP) pathways, and further analysis of *CPEB1* and *COLEC12* expression in cell lines, single cells and other cancers showed that they were strongly correlated with the survival and prognosis of patients with BC. This result suggested that patients with T2DM presenting the downregulation of *CPEB1* and *COLEC12* might have a higher risk of developing BC. Overall, our work revealed that high expression of CYPs in patients with T2DM might be a susceptibility factor for BC and identified novel gene candidates and immune features that are promising targets for immunotherapy in patients with BC.

## Graphical Abstract

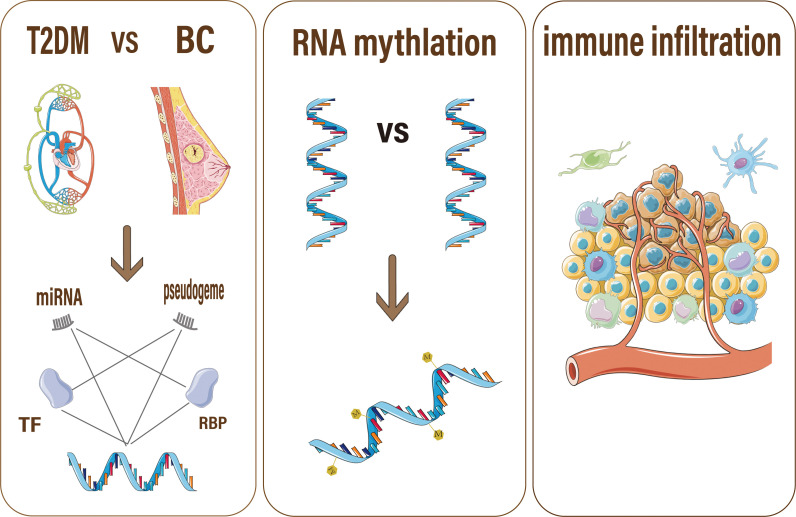

## Introduction

Type 2 diabetes mellitus (T2DM) is one of the most common chronic conditions worldwide, affecting males and females from all walks of life (1). Breast cancer (BC), the most prevalent malignancy in both women and men, is the 2nd leading cause of cancer-related death (2). Based on accumulating evidence, T2DM and BC are interrelated. Both are aging-related illnesses with a wide range of risk factors, including socioeconomic status, lifestyle choices, and body fat (3). Even after correcting for overweight/obesity, which is the main shared risk factor, several meta-analyses (4, 5) have indicated a pooled 15–20 percent increase in the incidence of BC among women with preexisting T2DM. Furthermore, a 10-year follow-up study found that diabetes/impaired glucose tolerance affects BC prognosis (6) and is positively correlated with BC-related death (7). In addition, a tumorigenic effect of hyperinsulinemia, insulin-like growth factors, and other hormones has been proposed as a causative link between T2DM and BC (8). However, the chemical mechanism(s) underlying the relationship between T2DM and BC remain unknown.

This study focuses on the shared mechanism of gene regulation between T2DM and BC. Gene regulation is mediated by a complex regulatory machinery. Disturbance of this precise machinery results in aberrant cell behaviors, which may cause cancer (9). One important step in gene regulation is mRNA expression (10). The fate of mature mRNA is influenced by noncoding RNAs (e.g., miRNAs) and RNA binding proteins (RBPs) as key determinants of posttranscriptional control (11). RBPs are defined as proteins capable of binding double- or single-stranded RNA, including mRNA and miRNA, and thereby influencing RNA fate (12). This interaction may, to some extent, explain why patients with T2DM have a higher risk of developing BC.

MiRNAs, a type of small noncoding RNA, regulate gene expression by binding to miRNA response elements (MREs) on target transcripts (13) and hence actively participate in cancer and diabetes (14). Atypical expression of certain miRNAs has been identified in the development and progression of several human malignancies (15). Several case–control studies and meta-analyses of European (16), Asian (17), Arab (18), and Jewish (19) communities have analyzed links between miRNA gene polymorphisms and the BC risk. The expression of miR-27a was reported to be significantly lower in samples from patients with BC presenting A/G or G/G genotypes than in samples from patients with A/A genotypes, implying that the A-to-G transition reduces mature miR-27a expression (20). Although transcription factors (TF) play important roles in initiating and regulating the transcription of mRNAs, miRNA expression is mediated by transcription TFs, according to previous research (21). Therefore, we built a TF-miRNA‒mRNA regulatory network in BC and T2DM to elucidate potential shared mechanisms of gene expression between the two diseases.

Notably, miRNA biogenesis is regulated by RNA-binding proteins (RBPs) (12). Pseudogenes preferentially bind RBPs and miRNAs, thus participating in the competing endogenous RNA (ceRNA) network for gene regulation (22). In addition, Pseudogenes might regulate their protein-coding counterparts *via* a ceRNA mechanism, participating in the pathological process of BC (23). In BC, cancer cells generally require increased levels of transcription and pre-RNA synthesis controlled by TFs, consequently increasing the cell’s dependence on RBPs (24). Mutations and epigenetic modifications may cause aberrant RBP expression in BC cells (25). Our study constructed a shared mRNA-RBP-pseudogene network regulating gene expression in T2DM and BC, revealing the mechanism underlying the higher prevalence of BC among patients with T2DM.

Importantly, ceRNAs are RNAs that mediate unique RNA–RNA interactions. Long noncoding RNAs (lncRNAs), pseudogenes, and mRNAs crosstalk by competitively binding to shared miRNAs, allowing them to perform their biological functions. Large-scale investigations have recently revealed that deregulation of ceRNAs may play a role in the progression of various malignancies, including BC (26). As a supplement to this finding, we investigated the ceRNA-related mechanisms of the mRNA‒RNA binding protein (RBP)-pseudogene network to reveal the precise process of gene regulation.

**Figure 1:**
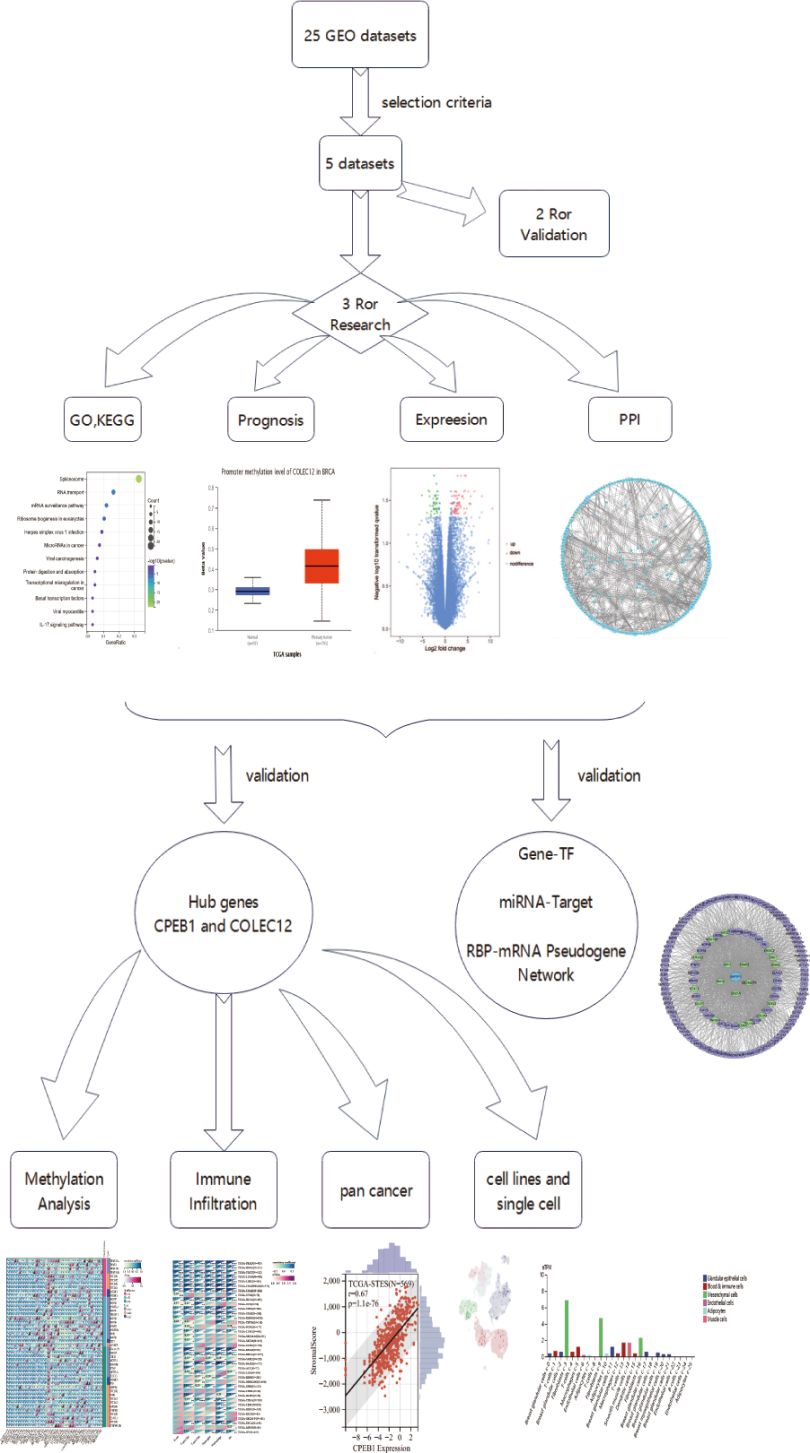
The flow chart for multiomics method.

In this article, we used multiomics analyses (genomic, proteomic, transcriptomic, epigenome (methylation), and immune cell infiltration analyses, **Figure 1** showed the flaw chart) to examine coexpressed hub genes and miRNAs in individuals with diabetes and BC, as well as key functions and pathways. We then focused on TF-miRNA‒mRNA networks and mRNA-RBP-pseudogene networks to determine the shared mechanisms and gene expression features of T2DM and BC. Surprisingly, our findings suggest that cytochrome (CYP)-related biological processes are critical in BC and T2DM. Treating cancer with specific agents while disregarding metabolic dysregulation may lead to therapeutic resistance in the tumor. The immunotherapy that links systemic metabolism to cancer will enable researchers to tailor agents specific for BC targets with metabolic dysfunction (27). We provide insights into analyzing immune microenvironments and gene regulation features, indicating that both *CPEB1* and *COLEC12* are promising targets for immunotherapy of patients with diabetes who are diagnosed with BC.

## Results

### GEO information and identification of DE genes

The three GEO datasets, GSE60436, GSE17907, and GSE101931, were chosen among the 25 datasets based on the following criteria: 1. include datasets produced within 10 years, 2. only includes datasets of *Homo sapiens*, 3. include datasets use similar sequencing methods, 4. exclude datasets of knock-out or overexpressed genes, and 5. exclude datasets without detailed descriptions. **Table S1** summarizes the information from the three datasets, such as the GSE number, detection systems, samples, and RNA sources. These datasets were then used in the differentially expressed gene (DEG) analysis. After identifying 2326 differentially expressed (DE) mRNAs in patients with BC, 2653 DE mRNAs in patients with T2DM, and 22 DE miRNAs in patients with T2DM, we extracted 200 verified DE miRNAs in BC from miRCancer and validated them using data from published articles (**Table S3**). **Table S2** contains information on these hub genes.

### Common gene signatures in BC and T2DM

**Figure 2:**
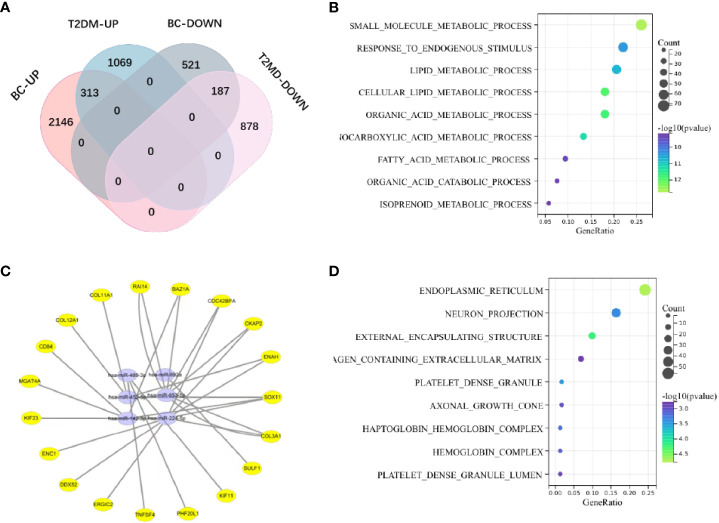
**(A)** The shared genes of T2DM and BC. **(B)** KEGG pathway analysis of coupregulated genes in T2DM and BC. **(C)** The miRNA‒mRNA regulatory network. Yellow circles represent mRNAs, and purple circles represent miRNAs. **(D)** Analysis of BP terms for coupregulated genes in T2DM and BC.

Among the evaluated GEO datasets, 427 overlapping DE mRNAs were detected in GSE60436 and GSE17907, comprising 167 downregulated and 231 upregulated genes, and the upregulated mRNAs were characterized as Gene Group 1 (GG1), while the downregulated mRNAs were defined as Gene Group 2 (GG2). The following shared downregulated DE miRNAs were selected by analyzing the data from HMDD and the GSE10197 profile (**Figure S1**): hsa-miR-224-5p, hsa-miR-452-5p, hsa-miR-892a, hsa-miR-653-5p, hsa-miR-489-3p, and hsa-miR-142-3p. Using a Venn diagram, the putative DE miRNAs generated from the two datasets were intersected (**Figure 2A**). All intersecting DE miRNAs are shown in **Table S1**.

### Biological analysis and functional annotation of the DE mRNAs

**Figure 3:**
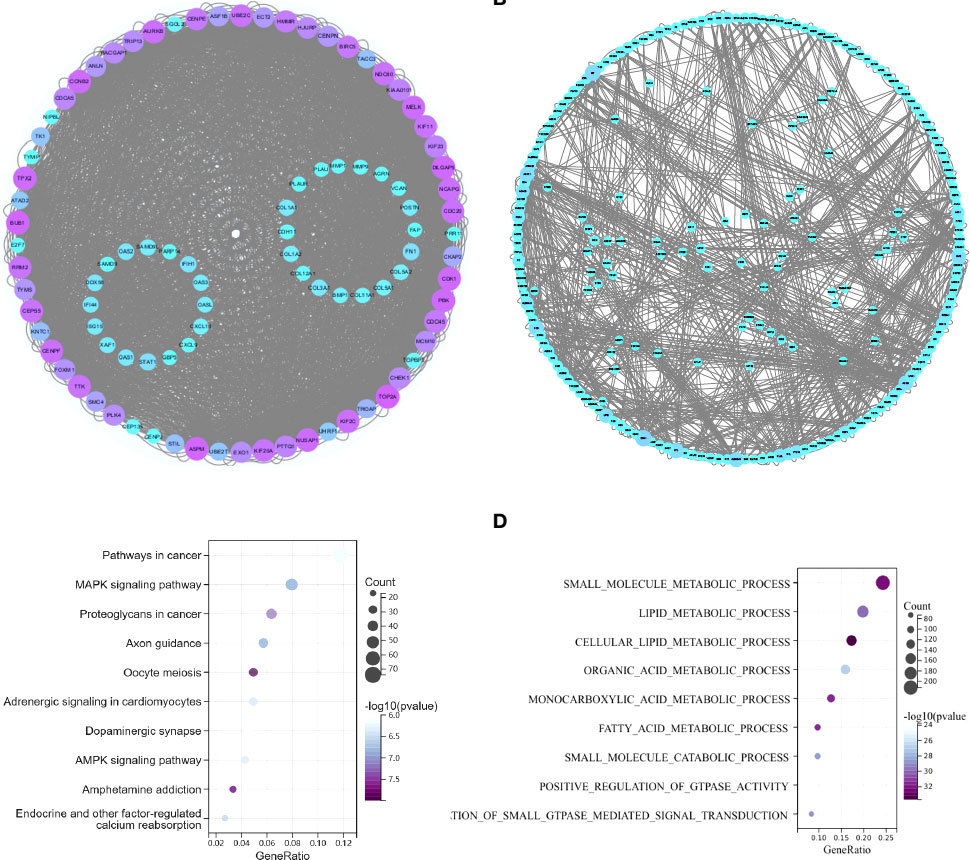
**(A)** PPI network of coupregulated genes in T2DM and BC. **(B)** PPI network of codownregulated genes in T2DM and BC. **(C)** KEGG pathway enrichment analysis of common miRNA target genes in BC and T2DM. **(D)** GO enrichment analysis of common miRNA target genes in BC and T2DM.

We investigated the probable functions of GG1 and GG2 using the R package “clusterprofiler” to conduct Gene Ontology (GO) and Kyoto Encyclopedia of Genes and Genomes (KEGG) enrichment analyses. “Cellular metal ion homeostasis”, “calcium ion homeostasis”, and “divalent inorganic cation homeostasis” were the top three enriched GO biological process (BP) terms. “Drug metabolism-cytochrome P450” and “metabolism of xenobiotics *via* cytochrome P450” accounted for 17% of the overall enriched GO terms and were related to 14 genes (**Figures 2B, D**, **Figure S2**), revealing that the P450 pathway is essential in both T2DM and BC. **Figure S3** depicts the GO/KEGG enrichment analyses of GG2. We explored the correlations between genes in GG1 and GG2 by generating protein‒protein interaction (PPI) networks using data from the STRING database (http://string.embl.de/) and visualized them using Cytoscape (**Figures 3A, B**).

### Analysis of differentially expressed genes in a validated cohort of patients with BC and T2DM

We analyzed DEGs in the GSE156993 and GSE45498 datasets to confirm our findings. Two hundred forty-six DEGs were identified in GSE45498, comprising 57 upregulated genes and 179 downregulated genes. Overall, 753 DEGs were identified in GSE156993, comprising 426 upregulated genes and 327 downregulated genes. Hierarchical clustering analysis of the DEGs distinguished between the case and control groups (**Figure S4**). In GSE156993, 15 hub genes among the 19 chosen genes were discovered, and all 19 genes were downregulated in GSE45498. We further identified the relationship between the 19 selected genes and BC and T2DM in DisGeNET and MalaCards, and we obtained relevant information for each gene, except *ERGIC2* and *DDX52*. Although *ERGIC2* and *DDX52* play important roles in the TF-miRNA‒mRNA network and mRNA-RBP-pseudogene network, the functions of *ERGIC2* and *DDX52* in BC and T2DM need further research. The 6 downregulated miRNAs were further validated in GSE160310, and the data were visualized using the R package eulerr (**Figure S5**).

### Identification and analysis of common miRNA target genes in BC and T2DM

According to 6 databases (miRWalk V2.0, mirDIP, miRSystem, miRDB, miRCancer and miRTarBase), 1543 mRNAs were associated with 6 shared downregulated miRNAs in BC and T2DM (Supplementary Data 1). The 1543 mRNAs were then investigated further. The top three enriched pathways were “route in cancer”, “MAPK signaling pathway”, and “proteoglycans in cancer”, as illustrated in the bubble charts (**Figures 3C, D**). Regarding enriched BP terms, “small molecule metabolic process”, “lipid metabolic process” and “cellular lipid metabolic process” were the three most highly enriched terms. Detailed information on the enriched pathways is listed in **Table S4**.

### The common TF-miRNA‒mRNA network in T2DM and BC

**Figure 4:**
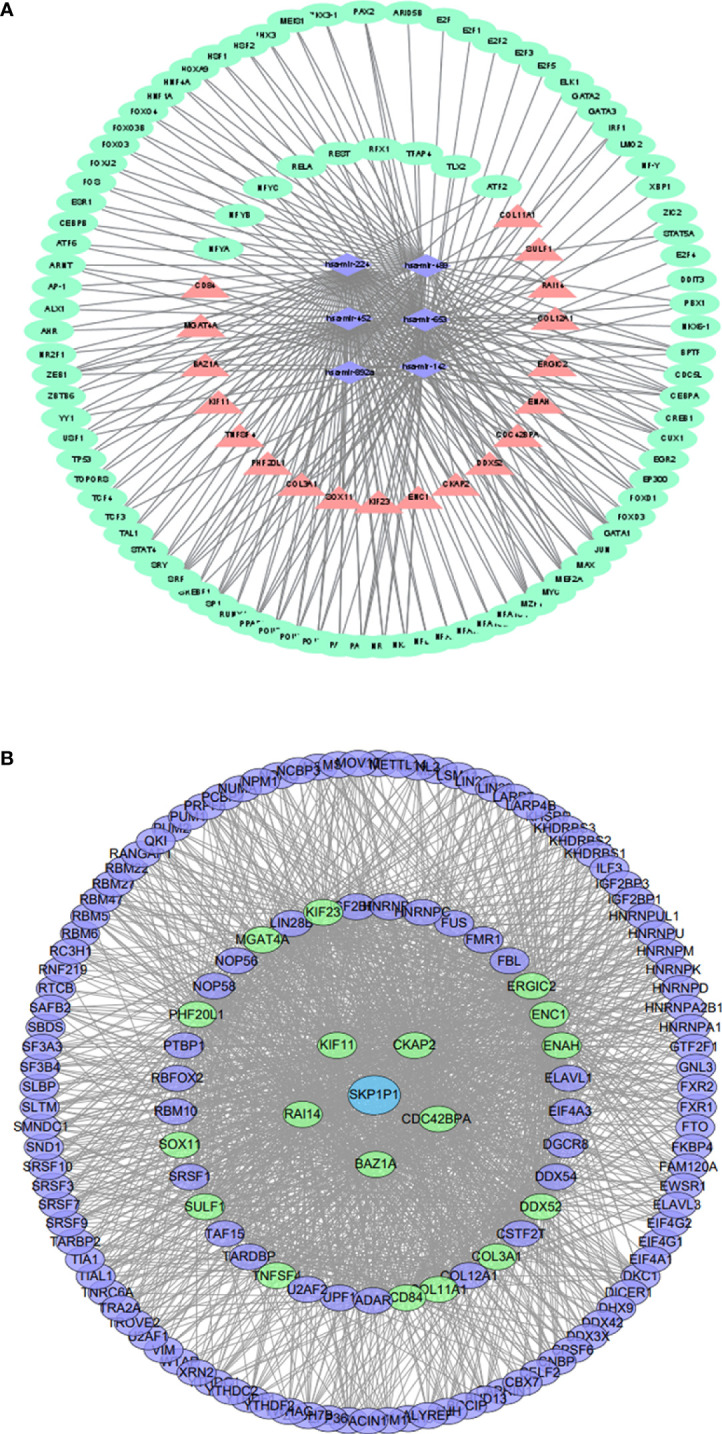
**(A)** TF-miRNA–mRNA regulatory network. Pink triangles represent mRNAs,purple diamonds represent miRNAs, and green circles represent shared genes. **(B)** The RBP–pseudogene-mRNA regulatory network. Blue circles represent pseudogenes, purple circles represent RBPs, and green circles represent mRNAs.

We constructed a network based on the 19 hub mRNAs, the interactions between DE mRNAs (GEO profile) and the target genes of the 6 hub DE miRNAs (confirmed in the 6 databases) in BC and T2DM. According to the relationship between the target genes and miRNAs derived from these online datasets, we constructed a miRNA‒mRNA network including 25 nodes, 6 miRNAs and 19 mRNAs, as shown in **Figure 2C**. Furthermore, we collected 236 TFs from TransmiR v2.0, and then a TF-miRNA‒mRNA network was constructed, including 261 nodes, 6 miRNAs, 19 mRNAs, 236 TFs and 255 edges (**Figure 4A**). The details of the nodes and interactions are listed in **Table S4**.

### Construction and functional annotation of the crosstalk between the hub mRNAs, RBPs and pseudogenes in BC and T2DM

As RBPs (RNA binding proteins) bind to mRNA while pseudogenes might bind to RBPs (22), we searched and downloaded the mRNA/RBP pairs and RBP/pseudogene pairs for the 19 hub mRNAs selected from the bioinformatics analysis of BC and T2DM using StarBase. According to the relationship between target genes provided by the online dataset, we constructed an RBP-mRNA-pseudogene network, including 147 nodes, 127 RBPs, 1 pseudogene, 19 mRNAs, and 1485 edges. The details of the nodes and interactions are listed in **Table S5**, and the network is shown in **Figure 4B**. Only one pseudogene, S-phase kinase associated protein 1 pseudogene 1 (SKP1P1), was included in the network, and it exhibited an experimental correlation with 38 important RBPs. Furthermore, we performed BP GO/KEGG enrichment analyses of the RBPs with the clusterProfiler R package. The top three significantly enriched BP GO/KEGG terms were “mRNA metabolic process”, “RNA processing” and “spliceosome” (**Figure S6**).

### Comprehensive analysis of the 2 shared hub DE mRNAs in T2DM and BC

**Figure 5:**
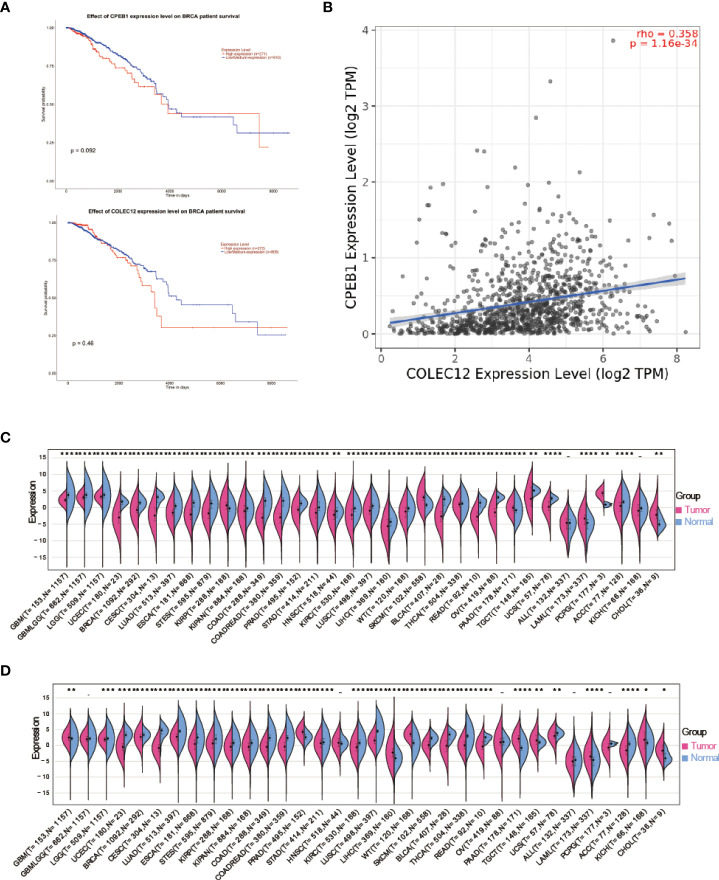
**(A)** Survival analysis of CPEB1 expression in patients with BC. **(B)** Survival analysis of COLEC12 expression in patients with BC. **(C)** Pancancer analysis of CPEB1 expression in tumors and normal tissues. **(D)** Pancancer analysis of COLEC12 expression in tumors and normal tissues. * in the picture indicates the significance of results, * equals to <0.05; ** equals to <0.01; *** equals to <0.001; **** equals to <0.0001.

We performed a Kaplan–Meier survival analysis of every mRNA to determine which mRNAs play key roles in RBP-mRNA-pseudogene and TF-miRNA‒mRNA networks. According to the results, *CPEB1* and *COLEC12* were substantially related to a better prognosis of BC (p<0.05), but the remaining upregulated mRNAs in both patients with BC and T2DM had no relationship with the survival of patients with BC (**Figure 5A** and **Figure S7**). Moreover, the expression of *CPEB1* and *COLEC12* was somewhat correlated in BC (**Figure 5B**). The PPI network also indicated that proteins encoded by *CPEB1* and *COLEC12* interacted with each other (**Figure 3B**). We further analyzed *CPEB1* and *COLEC12*, which are downstream target genes of hsa-miR-452-5p, using the methods described below to obtain a better understanding of their biological functions. In the expression analysis, we observed significant *CPEB1* upregulation in 6 tumors, including pancreatic adenocarcinoma (PAAD) (tumor: -0.02 ± 1.29, normal tissue: -0.96 ± 1.27, p=4.1e-14), and significant downregulation in 26 tumors, including BC (tumor: -0.74 ± 1.47, normal tissue: 1.37 ± 0.94, p=9.4e-96) (**Figure 5C**). As shown in **Figure 5D**, significant upregulation of *COLEC12* was observed in 9 tumors, including liver hepatocellular carcinoma (LIHC) (tumor: - 2.15 ± 2.15 ± 2.14, normal tissue: 4.08 ± 1.08 ± 1.77, p = 7.6e-22) and PAAD (tumor: 1.95 ± 1.85, normal tissue: - 0.95 ± 1.85, normal: - 0.84 ± 1.7e-36), and significant downregulation was detected in 20 tumors, including BC (tumor: 2.59± 1.56, normal tissue: 3.29 ± 0.90, p=3.0e-14) and colon adenocarcinoma (COAD)/rectal adenocarcinoma (READ) (tumor: -0.35 ± 1.91, normal tissue: 2.25 ± 1.74, p=2.4e-70). However, the cBioPortal algorithm showed that *CPEB1* expression was altered in 22 (2.03%) of 1084 patients, while *COLEC12* expression was altered in 14 (1.29%) of 1084 patients with BC.

### Analysis of *CPEB1* and *COLEC12* RNA methylation and differential expression in cell lines and single cells

**Figure 6:**
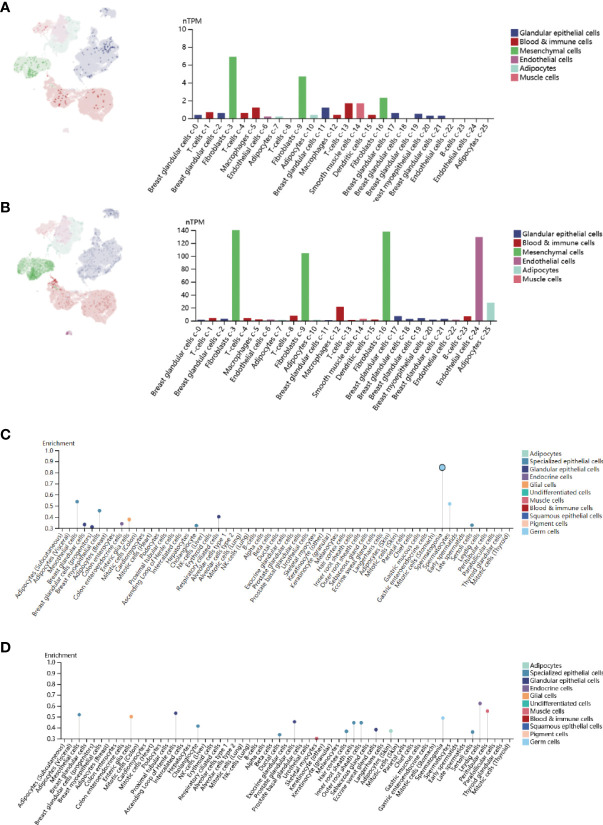
**(A)** Single-cell enrichment analysis of CPEB1 expression in breast tissue. **(B)** Single-cell enrichment analysis of COLEC12 in breast tissue. **(C)** Cell line enrichment analysis of CPEB1. **(D)** Cell line enrichment analysis of COLEC12.

**Figure 7:**
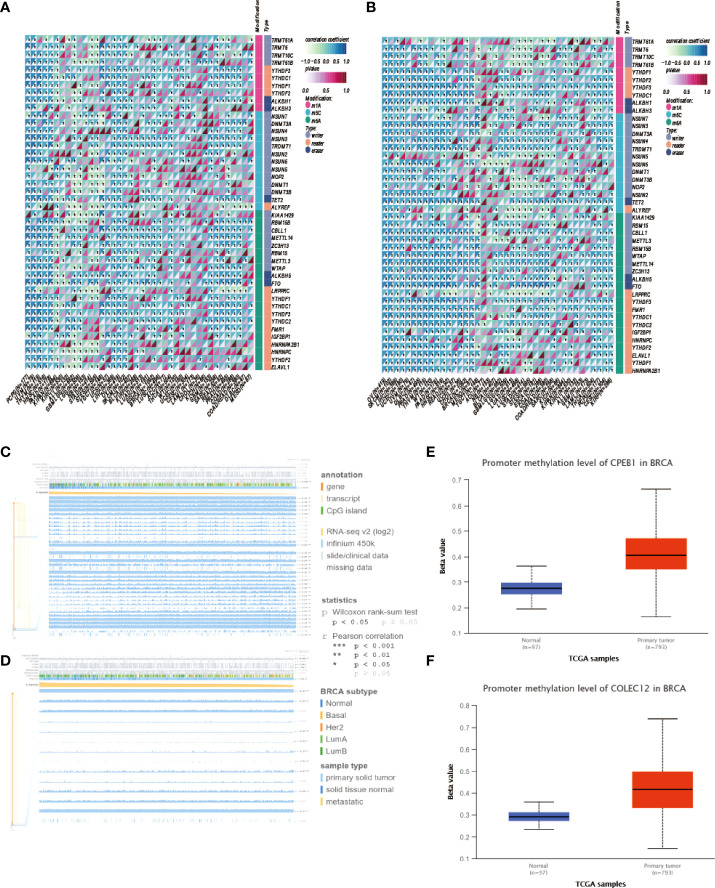
**(A)** Analysis of m1A, m5C and m6A modifications of the CPEB1 mRNA in cancers. **(B)** Analysis of the m1A, m5C and m6A modifications of the CPEB1 mRNA in cancers. **(C)** Analysis of the locations of CpG islands in CPEB1 in BC. **(D)** Analysis of COLEC12 methylation in breast cancer. **(E)** Level of CPEB1 promoter methylation in BC. **(F)** Level of COLEC12 promoter methylation in BC.

Gene expression is a stochastic process, with random alterations in transcription and translation leading to variations between cells at the mRNA and protein levels, especially in the immune system (28). Using the Human Protein Atlas (HPA), we investigated the *CPEB1* and *COLEC12* mRNA and protein levels in cell lines and single cells from various tissues (**Figures 6A–D**). *CPEB1* was expressed at high level in mesenchymal cell lines, while *COLEC12* was expressed at high levels in the brain, and its expression was lower in most single cells, except glial cells. N6-methyladenosine (m6A) is a reversible mRNA modification that has been shown to play important roles in breast cancer (29). Thus, we analyzed the N6-methyladenosine (m6A), N1-methyladenosine (m1A), and N6-methylcytosine (m6C) methylation of the *CPEB1* (**Figure 7A**) and *COLEC12* mRNAs (**Figure 7B**) in various cancers, and we discovered that the m6A modification of *CPEB1* displayed a stronger correlation with BC than the m5A and m1A modifications of *CPEB1*, and similar results were obtained for *YTHDF1* and *YTHDC2*. *COLEC12* exhibited a stronger association with the m1A and m6A modifications than with the m5C modification in BC. Finally, in each tumor, we utilized R software (version 4.0.1) to discern the difference in expression between normal and malignant samples. In 34 malignancies, the significance of differences in expression was determined using unpaired Wilcoxon rank sum and signed rank tests. We detected substantial upregulation in 9 tumors and significant downregulation in 20 tumors, including BC (tumor: 2.591.56, normal tissue: 3.329.90, p=3.0e-14).

### Relationship between methylation and *CPEB1*/*COLEC12* expression in BC

*CPEB1* and *COLEC12* were substantially methylated in BC tissue, according to the UALCAN algorithm (**Figures 7E, F**). Using the MEXPRESS algorithm, we investigated the link between *CPEB1*/*COLEC12* methylation and clinical data. We discovered relationships between substantial *CPEB1* and *COLEC12* methylation and a variety of clinical variables, including lymphocyte infiltration, PAM50 subtype, menopausal status, ER status, PR status, and HER2 status. **Figures 7C, D** show that *CPEB1* was methylated at various locations, including cg01776825, cg24689264, cg26565719, and cg07624612 (r = 0.368, 0.326, 0.409, and 0.423, respectively), whereas *COLEC12* was methylated at cg15630598, cg25570929, and cg21067023 (r = 0.169, 0.345, and 0, respectively). We assessed the relationship between the methylation of *COLEC12* sites (cg10737455, cg24475272, cg14201545 and cg12817260) and the methylation of *CPEB1* sites (cg26728382, cg05329960, cg14090920, cg00254888 and cg19630242) with clinical characteristics of patients using the MethSurv algorithm.

### Relationship between immune cell infiltration and *CPEB1* and *COLEC12* expression in BC and other cancers

**figure 8:**
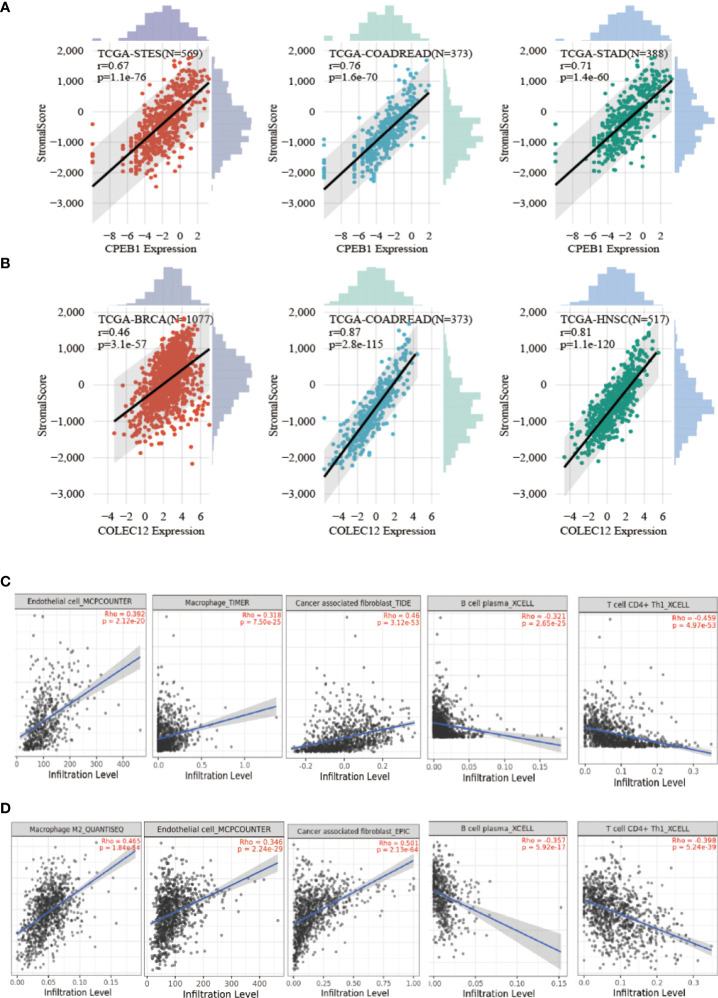
Analysis of CPEB1 and COLEC12 expression and immune cell infiltration. **(A)** Analysis of CPEB1 expression and immune cell infiltration in BC, colon adenocarcinoma, and ovarian serous cystadenocarcinoma. **(B)** Analysis of COLEC12 expression and immune cell infiltration in BC, colon adenocarcinoma, and ovarian serous cystadenocarcinoma. **(C)** Correlation of infiltrating immune cells and CPEB1 expression in BC **(D)** Correlation of infiltration of immune cells and COLEC12 expression in BC.

**Figure 9:**
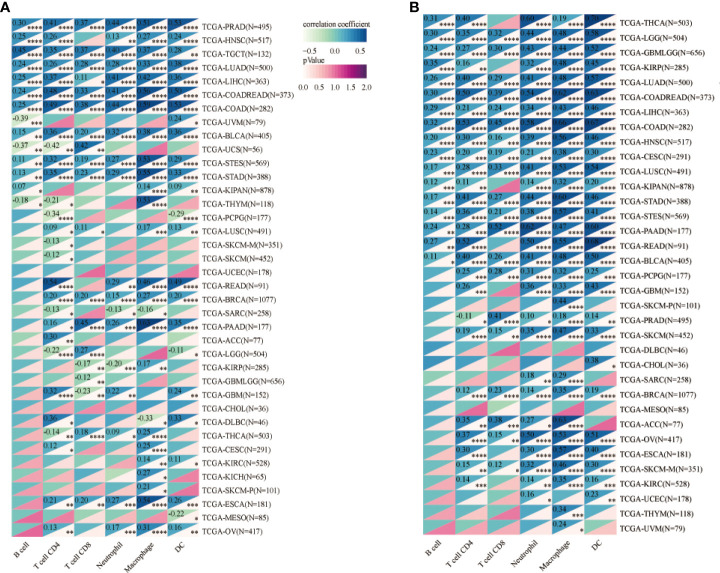
**(A)** Analysis of CPEB1 expression and immune cell infiltration in cancers. **(B)** Analysis of COLEC12 expression and immune cell infiltration in cancers. * in the picture indicates the significance of results, * equals to <0.05; ** equals to <0.01; *** equals to <0.001; **** equals to <0.0001.

We investigated the involvement of *CPEB1* and *COLEC12* in immune cell infiltration in BC using the TIMER platform to assess the link between DEGs and immune cell infiltration (**Figures 8C, D**). The strongest correlations with *CPEB1* expression were observed for cancer-associated fibroblasts (Cor = 0.460, p = 3.57E-50), endothelial cells (Cor = 0.380, p = 3.15E-33), macrophages (Cor = 0.318, p = 5.72E-23), CD4+ T cells (Cor = -0.460, p =4.55E-50) and natural killer (NK) T cells (Cor = -0.321, p = 2.20E-). The strongest positive correlations with *COLEC12* expression were observed for cancer-associated fibroblasts (Cor = 0.501, p = 4.86E-62) and macrophages (Cor = 0.465, p = 2.28E-52), whereas the strongest negative correlations with *CPEB1* expression were observed for CD4+ T helper 1 (Th1) cells (Cor = -0.398, p =3.48E-37) and B cells (Cor = -0.343, p = 3.55E-27). We explored the correlations between *CPEB1* and *COLEC12* expression and biomarker genes in immune cells. The findings showed the strongest associations with cancer-associated fibroblasts and macrophages (**Table S6**). We acquired the levels of infiltrating immune cells in 10,180 tumor samples from 44 different tumor types in a pancancer investigation. We discovered that *CPEB1* gene expression was significantly correlated with immune cell infiltration in 27 cancer types: 19 of these cancer types showed significantly positive correlations, including BC (N=1077, R=0.37, p=7.0e-37), COAD/READ (N=373, R=0.76, p=1.6e-70), LIHC (N=363, R=0.41, p=4.6e-16), ovarian serous cystadenocarcinoma (OV) (N=417, R=0.19, p=1.1e-4), and uveal melanoma (UVM) (**Figures 8A, B** and **9A, B**). *COLEC12* expression was significantly correlated with immune cell infiltration in 39 cancer types; 38 of these cancer types showed considerable positive correlations, including BC (N=1077, R=0.46, p=3.1e-57), COAD/READ (N=373, R=0.87, p=2.8e-115), LIHC (N=363, R=0.23, p=1.4e-5), OV (N=417, R=0.68, p=5.3e-57), and PAAD (N=177, R= 0.86, p=4.4e-54).

## Discussion

The pathophysiology of BC remains unknown, and the co-occurrence of T2DM and BC has previously been established. However, few studies have focused on the genetic aspects of BC in patients with T2DM. In the present study, we performed several types of bioinformatics analyses to investigate the shared processes of T2DM and BC with the aim of identifying novel therapeutic targets. The discussion will be separated into 3 parts: 1. Correlations between T2DM and BC in drug metabolism; 2. Correlations between T2DM and BC in the molecular mechanism; and 3. Guidance for immunotherapy of BC.

### Drug metabolism

#### CYPs play a vital role in the development of BC and T2DM and are associated with the relationship between BC and T2DM

Cytochrome P450 (CYP450) is a hemoprotein superfamily that is critical for drug biotransformation (26). Specific CYP superfamily isoforms have been discovered in cancers (30), in which they are presumed to modulate the response to anticancer treatment (5). CYP450s are highly conserved across species, suggesting that, in addition to their role in xenobiotic metabolism, they may have broader physiological activities. The top three enriched KEGG terms shared between T2DM and BC DE mRNAs were “drug metabolism-cytochrome P450,” “metabolism of xenobiotics by cytochrome P450,” and “chemical carcinogenesis.” In addition, CYP4B1, CYP7B1, and CYP26B1 are upregulated in BC, and CYP4X1 and CYP26A1 are upregulated in both BC and T2DM. The aforementioned findings show a strong link between CYPs and the common mechanisms of T2DM and BC. A study designed to examine the expression profile of CYP450 enzymes in the Caucasian population with BC (31) discovered that the *CYP4X1* gene was overexpressed. This elevated expression most likely alters the responsiveness of various pathological diseases to agents that are CYP2S1 substrates (32). As a result, our findings suggest that several CYP enzymes, such as CYP7B1 and CYP26B1, may play comparable pathogenic roles in BC, with variances probably attributable to the racially diverse populations investigated in various studies. *CYP4V2* and *CYP1B1* expression were substantially increased in GSE60436 in our investigation (T2DM profile). Several studies have highlighted a role for CYP1B1 in tumor growth and treatment resistance, suggesting that CYP1B1 is a potential oncological therapeutic target (33, 34).

Numerous CYP1B1 inhibitors have been developed to overcome treatment resistance in several tumor cell lines, and this strategy is recognized as the main therapeutic paradigm to treat malignancy (27). In individuals with diabetes, CYP expression exhibits an isoform-specific pattern. This altered expression might be partially modified by insulin treatment. An expression quantitative trait loci (eQTL) laser capture microdissection (LCM) analysis of iT2DMts from phenotyped pancreatectomized patients (PPPs) identified that CYP4V2 is associated with the levels of glycated hemoglobin A1c (HbA1c) (35), which plays a key role in the management of diabetes. As a result, we hypothesize that CYP4V2 plays an important role in the pathogenic phase of T2DM. According to another study, reduced expression of hsa-miR-27b is one factor contributing to elevated expression of the CYP1B1 protein in malignant cells (36). Although the upregulation of *CYP1B1*, one of the target genes of the hub DE miRNAs in BC (hsa-miR-27b), in our BC profile is not unexpected, patients with diabetes presenting high CYP1B1 expression may be more likely to develop BC, as *CYP1B1* might be downregulated by hsa-miR-27b, which is expressed during BC development.

### Metformin in BC and T2DM

Metformin is often regarded as a “foundation treatment” for individuals newly diagnosed with T2DM. This reputation stems from its excellent glucose-lowering abilities, low cost, neutral effect on weight, generally favorable safety profile (particularly the absence of hypoglycemia as an adverse effect), and some evidence for cardioprotection (1). Despite its popularity, debate still exists about the precise mechanism of action of metformin, although most data indicate a primary role for a reduction in hepatic glucose production (described further by Rena et al. in an issue of Diabetologia) (30). Intriguingly, metformin was also shown to enhance the function of the immune system and increase the potency of cancer treatment, although the molecular mechanisms underlying these effects are not fully understood. The study also indicated that metformin might be utilized to prevent the formation of BC while enhancing the prognosis of BC immunotherapy (27). In addition, metformin suppresses MAPK signaling in patients with T2DM (37), while the MAPK/ERK signaling pathway is also critical for the proliferation of estrogen-independent BC cells (38). The dose of metformin used to treat patients with diabetes who are at risk of developing BC is being investigated, and our findings show that it has a strong correlation with CYPs.

In conclusion, drug metabolism involving CYPs and metformin might participate in the same pathological processes of T2DM and BC. CYPs might contribute to the mechanism by which metformin improves the prognosis of immunotherapy for patients with BC, while aberrant expression of CYPs caused by BC might in turn impair the effect of metformin.

### Molecular mechanism

#### Novel insights into the shared ceRNA network of BC and T2DM

According to previous studies, several known ceRNA interactors are involved in a variety of illnesses, including cancer. Identification of these interactions has altered our understanding of illnesses and provided new opportunities for studying disease processes. Previous ceRNA analyses have focused on the competitive interactions between two molecules and have disregarded the multiple competitive links in cancer. Here, we revealed RBP-pseudogene-mRNA interactions, built a competitive network, and proposed cancer biomarkers based on the interactions between RBPs, pseudogenes, and mRNAs in BC and T2DM.

Pseudogenes have been shown to influence regulatory systems in pancancer investigations (16). One example is the PTEN-PTENP1 (pseudogene) interaction, which has been implicated in prostate cancer18. FTH1-FTH1PX (X represents multiple pseudogenes) in prostate cancer (18), SUMO1-SUMO1P3 in gastric cancer (19), and ATP8A2-ATP8A2 in BC (16) are a few additional regulatory pseudogene‒gene interactions that have been verified. These examples provide persuasive evidence that pseudogene‒gene interactions may be employed directly as predictors of human cancer, but candidate interactions must be identified to properly exploit these associations. SKP1P1 was the only pseudogene identified in the mRNA-RBP-pseudogene network revealed in this study and has known regulatory relationships with several RBPs and mRNAs, suggesting that it may be a relevant regulatory gene in the ceRNA network of BC and T2DM. Our study also revealed an RBP-pseudogene-mRNA network, which will be beneficial for practical ceRNA-related research.

### Promising therapeutic targets for BC: *CPEB1* and *COLEC12*

*CPEB1* encodes a protein that interacts with a particular RNA sequence known as the cytoplasmic polyadenylation element. The encoded protein has cytoplasmic and nucleolar activities. It regulates mRNA translation and the processing of the 3’ untranslated region, and it may have a role in modulating cell proliferation in cancer. *In vivo*, lower *CPEB1* levels enhance BC cell metastasis to the lung, whereas ectopic expression of *CPEB1* substantially inhibits this process, implying that *CPEB1* might be a good prognostic factor for predicting human BC metastasis. Nonetheless, *CPEB1* deficiency has little effect on polarity in intestinal or kidney epithelial cells20, and as this process is presumed to be a crucial initial event, CPEB1 unlikely has much of an effect on metastasis in these cells. Furthermore, ectopic expression of a dominant-negative form of CPEB1 that does not induce polyadenylation limits the production of metadherin (MRDH), a metastasis-promoting factor, and reduces migration and tumor formation of glioblastoma cells. Due to inconsistencies in the aforementioned data, CPEB1 likely exerts diametrically opposing effects on metastasis depending on cell type. As a result, the overexpression of *CPEB1* in immune cells may be associated with a distinct biological mechanism that is relevant to patients with T2DM secondary to BC.

Collectin-12 (CL-12), also known as collectin placenta 1 (CL-P1), is a pattern recognition molecule (PRM) of the innate immune system that is encoded by the *COLEC12* gene (39) CL-12 was originally defined as a scavenger receptor C-type lectin (40) and is mainly expressed in cells that originate from the endothelium and macrophages (41). Moreover, CL-12 is suggested to be involved in leukocyte recruitment and cancer metastasis. Common polymorphisms in or near *COLEC12* have been linked to diabetic retinopathy in Chinese patients with T2DM (42). In our work, *CPEB1* and *COLEC12* were expressed at high levels in fibroblast-3 cells (**Figures 9A, B**), contributing to the construction of the tumor immune microenvironment. They were shown to be expressed at elevated levels in BC and to be target genes of hsa-miR-452-5p in both patients with BC and T2DM.

Many studies have shown that increased immune cell infiltration is related to better clinical outcomes and treatment responsiveness in patients with BC 4-11 (43–48). Daniel Winer and colleagues reported alterations in the intestinal immune system that may affect systemic immunity and glucose metabolism, contributing to obesity and diabetes pathogenesis (16) (49). In our study, *CPEB1* and *COLEC12* were strongly correlated with immune cell infiltration. Notably, both *CPEB1* and *COLEC12* were expressed at significantly higher levels in fibroblasts, an essential component of the tumor microenvironment. Based on accumulating evidence, aberrant m6A modifications are linked to the epigenetic control of the immune response in individuals with BC. Similarly, the m1A modification has been shown to improve translation efficiency (50–52). In the present study, *CPEB1* expression in BC was related to the m6A modification, whereas *COLEC12* expression was associated with both m1A and m6A modifications. Three distinct m6A modification patterns have been reported, and the infiltrating properties of cells in the tumor microenvironment correspond to the three immunological phenotypes, which include immune rejection, immune inflammation, and immune desert (53). The research findings described above suggest that the m6A modification of CPEB1/*COLEC12* and the m1A modification of *COLEC12* in BC exert significant effects on some immune responses.

In conclusion, T2DM and BC share some similar patterns of gene regulation. Based on the results obtained using multiomics approaches, including methylation, genomics, proteomics, immune cell infiltration, prognostic analysis and pancancer analysis, *CPEB1* and *COLEC12* are likely to be key drug targets and immunotherapy targets.

### New insights into immunotherapy for patients with T2DM diagnosed with BC

Immunotherapy acts mainly by harnessing immune cells within or outside the TME to specifically recognize and attack cancer cells (3). Immune checkpoint inhibitors (ICIs) are some of the main drugs used in immunotherapy. The use of ICIs—monoclonal antibodies targeting programmed death protein 1 (PD-1), programmed death protein 1 ligand (PD-L1) and CTLA-4—has yielded impressive results in many settings and is currently a cornerstone in cancer treatment, including BC (54). Nevertheless, metabolic side effects will likely be an important consequence of immunotherapy using ICIs for BC treatment (55). Multiple mechanisms, such as the overexpression of immune checkpoint molecules, loss of nutrients through vascular impairment and dysregulation of metabolic pathways, were recently shown to affect T cell recruitment and metabolic activities in the tumor microenvironment (TME) (56, 57) Preclinical models and human studies of immunotherapy suggested that the general pathology of common forms of diabetes, namely, insulin resistance (T2DM) and/or impaired insulin secretion, may be exacerbated by ICIs (58). Therefore, patients receiving cancer immunotherapy have an increased risk of diabetes.

The TME is very important in metabolic alterations caused by immunotherapy. In addition, the TME also determines tumor immunology, progression, metastasis, and the response to immunotherapy (59). The TME is profoundly immunosuppressive, which is a key factor explaining why most cancer therapies that operate (or in part operate) by stimulating immune cell actions against cancer continue to display limited clinical efficacy. At present, many immunotherapies exist to remove the obstruction of the TME, including the targeting of excessive immunoregulatory angiogenesis. Suppression of fibroblasts is also a novel approach. Outstanding clinical efficacy of the modulation of the TME to improve the response to immunotherapy is also being investigated. Fibroblasts, stromal cells in the TME associated with tumor formation and metastasis in BCs (60), are the most essential components of the TME. Through multiple pathways, activated fibroblasts promote tumor growth, angiogenesis, invasion and metastasis. Fibroblasts interact with tumor-infiltrating immune cells and other immune components within the tumor immune microenvironment (TIME), consequently shaping an immunosuppressive TME that enables cancer cells to evade surveillance of the immune system (61). Our research reveals that *CPEB1* and *COLEC12* are substantially upregulated in fibroblasts, according to analyses of cell lines and single cells using BC data. In-depth studies of the expression of CPEB1 and COLEC12 in fibroblasts and their roles in immune microenvironment interactions, particularly the complicated mechanisms connecting fibroblasts with immune cells, might provide novel strategies for BC immunotherapies in patients with diabetes. Although the long-term consequences of developing diabetes secondary to these new anticancer agents are poorly understood, our study provides solid evidence from multiomics analyses for these processes by exploring the shared immunological mechanism between T2DM and BC.

Clearly, our study has significant limitations as well. We collected information on miRNAs, RBPs, and pseudogene targets from StarBase; mRNAs were confirmed in online datasets and GEO datasets rather than experiments; and the miRNA targets were not exhaustive due to data recording limitations. Although the study suggests that CPY-related metabolism is important for shared pathological processes in T2DM and BC, cancer treatment for patients with BC to prevent metabolic dysfunction, the time of function, the amount of CYPs that are required and the complicated mechanisms underlying these processes still require further research and experiments. In addition, an immunotherapy is unavailable for patients with diabetes diagnosed with BC, and the application of potential targets still lacks clinical trials. Our study remains noteworthy, however, since the targets provide insights for future studies of patients with T2DM and BC.

In summary, we revealed a TF-miRNA‒mRNA network and an RBP-mRNA-pseudogene network from genome-wide transcriptome data using various bioinformatics analyses to comprehensively analyze the possible shared mechanisms of BC and T2DM. We revealed that the participation of some immune cells and the presence of some hub genes (miRNAs, mRNAs, pseudogenes, TFs and RBPs) in patients with T2DM might be essential susceptibility factors for BC and identified novel gene candidates that might be used as biomarkers or as potential therapeutic targets.

## Methods

### Downloading GEO datasets

We searched the GEO database for gene expression profiles in patients with BC and T2DM using the key words “breast cancer” and “breast carcinoma” or “diabetes mellitus” and “type 2 diabetes mellitus.” The retrieved datasets were filtered utilizing the criteria listed below. First, gene expression profiling studies had to include both cases and controls. Second, the sequencing cell source was peripheral blood mononuclear cells (PBMCs). Third, the proportion of female patients must be greater than 80%, ensuring that the research had practical application. Fourth, patient age ranged from 30 to 70 years, since T2DM is more common in elderly patients. Fifth, the studies had to include processed or raw data that could be reanalyzed. The author stated in the initial report of GSE60436 that the admission criteria for patient samples included a diagnosis of T2DM and HbA1c level ≤13%, which were suitable criteria for our research. Ultimately, the GEO datasets GSE101931, GSE60436 and GSE17907 were chosen. **Table S1** contains detailed information about the datasets utilized in this investigation. We searched for hub miRNAs of BC in articles published in the last ten years and collected data from miRCancer. **Table S5** contains detailed information on the identified miRNAs.

### Identification of DE miRNAs and DE mRNAs

Raw GSE101931, GSE17907, and GSE60436 data files were read using the oligo R package. The data were successively filtered, adjusted for the background, log base 2 transformed, and normalized. Gene symbols were acquired by probe conversion based on platform annotation information. If one gene symbol matched two or more probes, the mean expression level of these matching mRNAs or miRNAs was used to calculate the final expression level. We employed an R program for data processing and visualization (version 4.0.1). A moderated t test was used to compare the expression levels of mRNAs or miRNAs between groups in the datasets. A p value < 0.05 and | log2FC| > 1.5 were used as limits for determining statistical significance to evaluate the differential expression of mRNAs or miRNAs in the datasets. The mRNA expression levels of hub genes in BC and T2DM samples were statistically analyzed using a paired Student’s t test, and p values less than 0.05 were considered significant.

### PPI network generation and GO and KEGG analyses

We used the clusterProfiler R package (62) to perform GO and KEGG analyses of the functions of the selected genes. The GO analysis included BP, cellular component (CC), and molecular function (MF) terms. A p value of 0.05 was regarded to indicate considerable enrichment, and the BH technique was employed to modify the p value. Two PPI networks were created and analyzed with the STRING database to elucidate shared molecular processes underpinning T2DM and BC and obtain insights into the interactions of the shared 313 upregulated and 187 downregulated genes in individuals with T2DM and BC. Target genes in the PPI network served as nodes, the lines between two nodes denoted associated interactions, and the strength of an interaction was indicated by the color of the line. The hub genes, which were defined as genes that played essential roles in the network, were distinguished according to the following criterion: degree calculated by CytoHubba in Cytoscape. The corresponding interactions were visualized using Cytoscape (63).

### DEG analysis and validation of shared and distinct gene signatures

We replicated the DEG analysis with additional T2DM and BC datasets (GSE156993 and GSE45498) to confirm the shared and distinct genes involved in T2DM and BC. The R program limma (64) was used to analyze the DEGs between the case and control groups. A |log2(fold change)| greater than 0.56 and a p value of 0.05 were used as cutoff values. The expression patterns of the DEGs were determined using hierarchical clustering analysis and heatmaps. The overlapping DEGs in the T2DM and BC datasets were determined using the R tool eulerr. Furthermore, we utilized DisGeNET and MalaCards to identify 19 hub genes, which we used to establish a TF-miRNA‒mRNA network. DisGeNET is a research platform that consists of one of the largest publicly released libraries of genes and variants identified in human diseases. DisGeNET incorporates information from expert-curated repositories, genome-wide association studies (GWAS) libraries, animal models, and the scientific literature. Several innovative measures are also employed to aid in the prioritization of genotype–phenotype interactions. MalaCards is a combined human disease database with broad clinical and genetic annotations and a systematic search ability (65). Using the R package limma, GSE160310 was utilized to verify the DE miRNAs in T2DM, and the cutoff values were |log2(fold change)| > 0.6 and p value < 0.05. The findings from the enrichment analyses of the discovery and validation cohorts were compared to determine whether our analytical approach was reliable.

### Construction of the shared miRNA‒target gene-TF network

Investigating miRNA target genes is critical for understanding miRNA regulatory mechanisms and functions. We identified eight upregulated miRNAs and six downregulated miRNAs and then predicted the targets of the DEGs using six miRNA‒target tools: miRWalk V2.0 (65), mirDIP (66), miRSystem (67), miRDB (68), miRCancer (69), and miRTarBase (70). The overlapping results from the six databases were used to screen the miRNA targets. We extracted the shared genes in the target gene group from the database and the DEGs of these two diseases (BC and T2DM) by performing bioinformatics analyses. We predicted upstream TFs of the 6 hub miRNAs with TransmiR v2.0 (71), a public tool that integrates empirically proven TF-miRNA regulatory connections from publications. The intersection of comparable miRNA target genes and shared genes in T2DM and BC (based on a fold change in expression >2.5 and an FDR 0.05) was utilized to establish the miRNA–mRNA regulatory network, which was then visualized using Cytoscape (63).

### StarBase analysis and construction of the RBP–mRNA-pseudogene network

StarBase (72),a widely used open-source platform for analyzing ncRNA interactions *via* CLIP-seq, degradome-seq and RNA–RNA interactome data, was employed to investigate the associations between mRNA, RBP, and pseudogene expression. R < -0.1 and a p value < 0.05 were defined as the cutoff criteria for identifying the key mRNA-RBP pairs and RBP-pseudogene pairs to assess crosstalk between the shared hub mRNAs of BC and T2DM pseudogenes and RBPs. Subsequently, the RBP-pseudogene-mRNA network was built with Cytoscape. We used the clusterProfiler R package to conduct GO and KEGG analyses of the potential genes and further investigate their functions. BP, CC, and MF terms were assessed in the GO analysis. A p value of 0.05 was regarded to indicate considerable enrichment, and the BH technique was employed to modify the P value.

### Analysis of the correlation between immune cell infiltration and *CPEB1* and *COLEC12* expression

We investigated the expression of *CPEB1* and *COLEC12* in malignant tumors, as well as the relationship between their expression patterns and the levels of infiltrating immune cells with TIMER (73), a resource that enables a systematic analysis of the quantities of infiltrating immune cells in various cancers. We obtained a unified and standardized pancancer dataset from UCSC (74), TCGA, TARGET, and GTEx (PANCAN, N=19131, G=60499) and retrieved ENSG00000214575 (*CPEB1*) and ENSG00000158270 (*COLEC12*) gene expression data for each sample. We next filtered the sample sources: primary blood-derived cancer - peripheral blood (acute myeloid leukemia (LAML)); primary tumor – metastasis of skin cutaneous melanoma (SKCM); primary blood-derived cancer - bone marrow; primary solid tumor; and recurrent blood-derived cancer - bone marrow. Data from these samples were further log2(x+0.001) transformed, and the gene expression profile of each tumor was extracted and mapped. The R software program ESTIMATE was then applied to determine the gene symbols and gene expression in each tumor. Stromal, immune, and ESTIMATE scores were calculated for each patient. In addition, we utilized TIMER to analyze B cell, CD4+ T-cell, CD8+ T-cell, neutrophil, macrophage, and dendritic cell (DC) infiltration in each tumor sample from each patient based on the gene expression score.

### Methylation analysis of *CPEB1* and *COLEC12*

We acquired a UCSC unified and standardized pancancer dataset including TCGA, TARGET, and GTEx data (PANCAN, N=19131, G=60499), and we further extracted ENSG00000214575 (*CPEB1*) and ENSG00000158270 (*COLEC12*) data from it. Data regarding gene modifications and three types of RNA modifications (m1A (10), m5C (13), and m6A (21)) in each sample were extracted, and we subsequently screened the following sample sources: primary solid tumor; primary tumor; primary blood-derived cancer - bone marrow; and primary blood-derived cancer - peripheral blood. In addition to filtering all normal samples, we log2(x+0.001) transformed each expression value and calculated the Pearson correlation coefficients between ENSG00000214575 (*CPEB1*)/ENSG00000158270 (*COLEC12*) expression and the expression of immune pathway signature genes. The UALCAN algorithm (75) is a data-mining platform that has been used to assess the methylation of DE mRNAs in tumors. The UALCAN algorithm was used in this study to examine *CPEB1*/*COLEC12* methylation in BC and normal tissues. MEXPRESS is a data visualization application that is used to depict TCGA expression data and the link between methylation and clinical data.

### Pancancer analysis of *CPEB1* and *COLEC12* expression

Using the HPA algorithm, we evaluated the *CPEB1* and *COLEC12* mRNA and protein levels in diverse organs (**Figures S4**, **S5**). We employed RNA sequencing data to assess *CPEB1* and *COLEC12* expression in cell lines and single cells. Next, in each tumor, we utilized R software (version 4.0.1) to determine the difference in expression between normal and malignant samples. In 34 cancers, the significance of the difference was determined using unpaired Wilcoxon rank sum and signed rank tests.

### HPA and cBioPortal analyses

HPA was established in 2003 with the goal of mapping all human proteins in cells, tissues, and organs. Using this resource, we determined the protein and RNA expression levels of *CPEB1* and *COLEC12* in multiple cancer tissues (76). cBioPortal is an online source for visualizing cancer genomics data that provides information on somatic mutations, changes in copy number, and mRNA expression (77). We used cBioPortal to assess alterations in the *CPEB1* and *COLEC12* genes in BC in this study.

## Data availability statement

The datasets presented in this study can be found in online repositories. The names of the repository/repositories and accession number(s) can be found in the article/**Supplementary Material**.

## Author contributions

FM and YG conceived the study, critically reviewed the intellectual content of the manuscript and made substantive revisions to the important contents of the manuscript. WT was the major contributor to the research and the writing of the manuscript. GW participated in the drawing of the graphical abstract and the insertion of references. HL; CY; ZH; GX and YX provided suggestions and technical support and revised important sections of the manuscript. All authors contributed to the article and approved the submitted version.

## Conflict of interest

The authors declare that the research was conducted in the absence of any commercial or financial relationships that could be construed as a potential conflict of interest.

## Publisher’s note

All claims expressed in this article are solely those of the authors and do not necessarily represent those of their affiliated organizations, or those of the publisher, the editors and the reviewers. Any product that may be evaluated in this article, or claim that may be made by its manufacturer, is not guaranteed or endorsed by the publisher.
