# Supplementary material for: Exploration of shared TF-miRNA‒mRNA and mRNA-RBP-pseudogene networks in type 2 diabetes mellitus and breast cancer
Source: Front Immunol. 2022 Sep 5;13:915017. doi: 10.3389/fimmu.2022.915017 (PMC9484524; doi:10.3389/fimmu.2022.915017)
Supplement: Supplementary file 1 [file DataSheet_1.pdf]

Table S1. GEO information of datasets and Validated Cohort

GEO	Platform	Tissue (Homo sapiens)	Sample (Number) Total	Con	BC/T2DM	Experiment type	Attribute	Author	Disease
GEO:GSE60436	GPL6884	retina	9	3	6	array	test	Ishikawa K, Yoshida S	T2DM
GEO:GSE101931	GPL10558	blood	22	8	14	array	test	Hubal MJ, Gold L	T2DM
GEO:GSE17907	GPL570	breast	198	33	165	array	test	Sircoulomb F, Finetti P	BC
GEO:GSE160310	GPL20301	blood	80	20	60	array	test	Hubal MJ, Gold L	T2DM
GEO:GSE156993	GPL570	peripheral blood mononuclear cells	30	12	18	array	test	Scarel-Caminaga RM, Corbi SC	T2DM
GEO:GSE45498	GPL16299	lymph node	228	59	169	array	twst	Cascione L, Gasparini P	BC

Table S4 KEGG analysis of common miRNA target genes in BC and T2DM

Description (KEGG)	ratio
Oocyte meiosis	31/630
Amphetamine addiction	21/630
Proteoglycans in cancer	40/630
MAPK signaling pathway	50/630
Axon guidance	36/630
Endocrine and other factor-regulated calcium reabsorption	17/630
Adrenergic signaling in cardiomyocytes	31/630
AMPK signaling pathway	27/630
Pathways in cancer	74/630
Dopaminergic synapse	28/630

Table S5 Downregulated hub miRNA in T2DM

downregulated	logFC	AveExpr	t	P-value	adj.Pval	B
**hsa-miR-224**	-3.3758	2.736	-6.0788	2.55E-7	3.5879E-4	6.6861
**hsa-miR-452**	-2.9103	0.69813	-4.9004	1.3291E-5	0.0092313	3.1
**hsa-miR-892a**	-2.678	0.12931	-3.93	2.99546E-4	0.028566	0.23944
**hsa-miR-653**	-2.1482	0.45149	-4.3474	7.9887E-5	0.024503	1.4742
**hsa-miR-489**	-1.9919	0.45204	-3.6604	2.0586E-4	0.026457	0.61901
**hsa-miR-142**	-1.7172	-4.9583	-3.6684	4.5375E-4	0.036299	0.092357
**hsa-miR-218**	-1.5642	6.0648	-3.6181	2.9956E-4	0.028566	0.28102

Table S6 Significant CPEB1 and COLEC12 expression in immune cells

CPEB1	cancer	infiltrates	rho	p	adj.p
	BRCA (n=1100)	Cancer associated fibroblast_TIDE	0.460124	3.12E-53	3.57E-50
	BRCA (n=1100)	Endothelial cell_MCPCOUNTER	0.379955	1.72E-35	3.15E-33
	BRCA (n=1100)	Macrophage_TIMER	0.318374	7.50E-25	5.72E-23
	BRCA (n=1100)	T cell CD4+ Th1_XCELL	-0.45932	4.97E-53	4.55E-50
	BRCA (n=1100)	B cell plasma_XCELL	-0.32129	2.65E-25	2.20E-23
COLEC12	BRCA (n=1100)	Cancer associated fibroblast_EPIC	0.501407	2.13E-64	4.86E-62
	BRCA (n=1100)	Macrophage M2_QUANTISEQ	0.464955	1.84E-54	2.28E-52
	BRCA (n=1100)	Macrophage_TIMER	0.426623	3.13E-45	2.81E-43
	BRCA (n=1100)	T cell CD4+ Th1_XCELL	-0.39767	5.24E-39	3.48E-37
	BRCA (n=1100)	B cell plasma_XCELL	-0.34279	8.68E-29	3.55E-27
	BRCA (n=1100)	Mast cell_XCELL	0.352019	2.27E-30	9.72E-29

Supplemental figure and legends

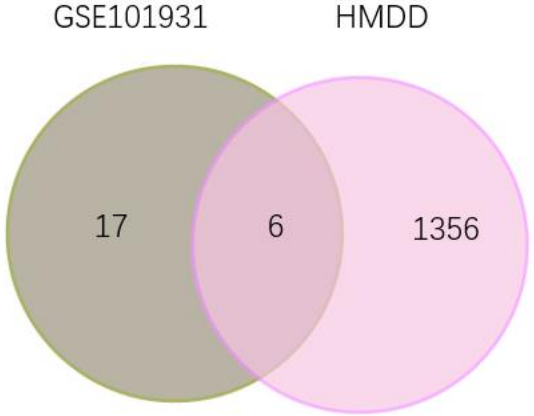

Fig. 1 validation analysis of down-regulated miRNAs

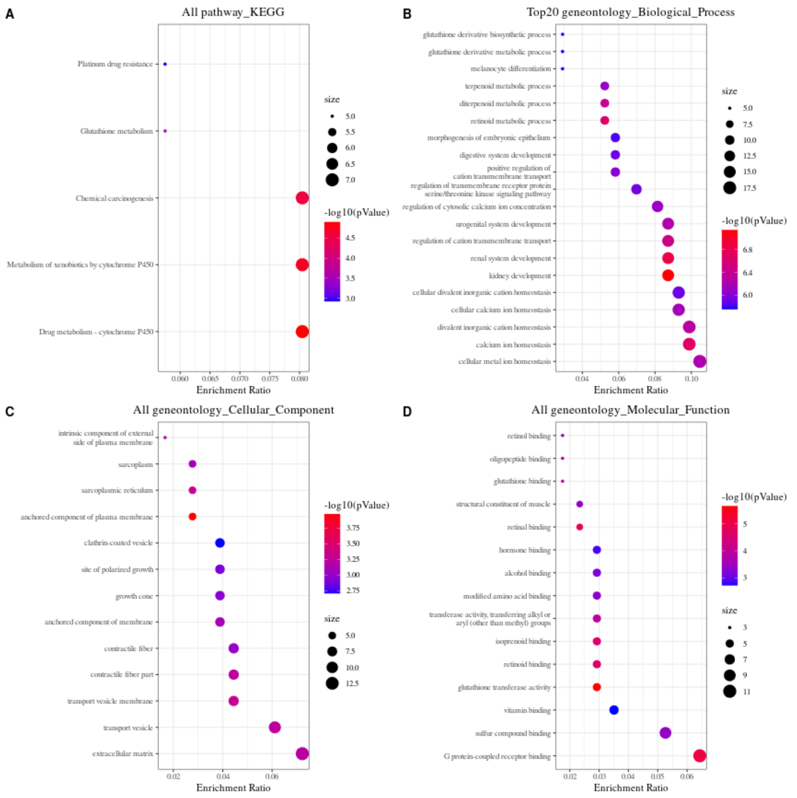

Fig. S 2 KEGG/GO enrichment analysis of co-upregulated genes: (A) KEGG pathway analysis (B) biological process analysis (C) cellular component snslysis (D) molecular funtion analysis

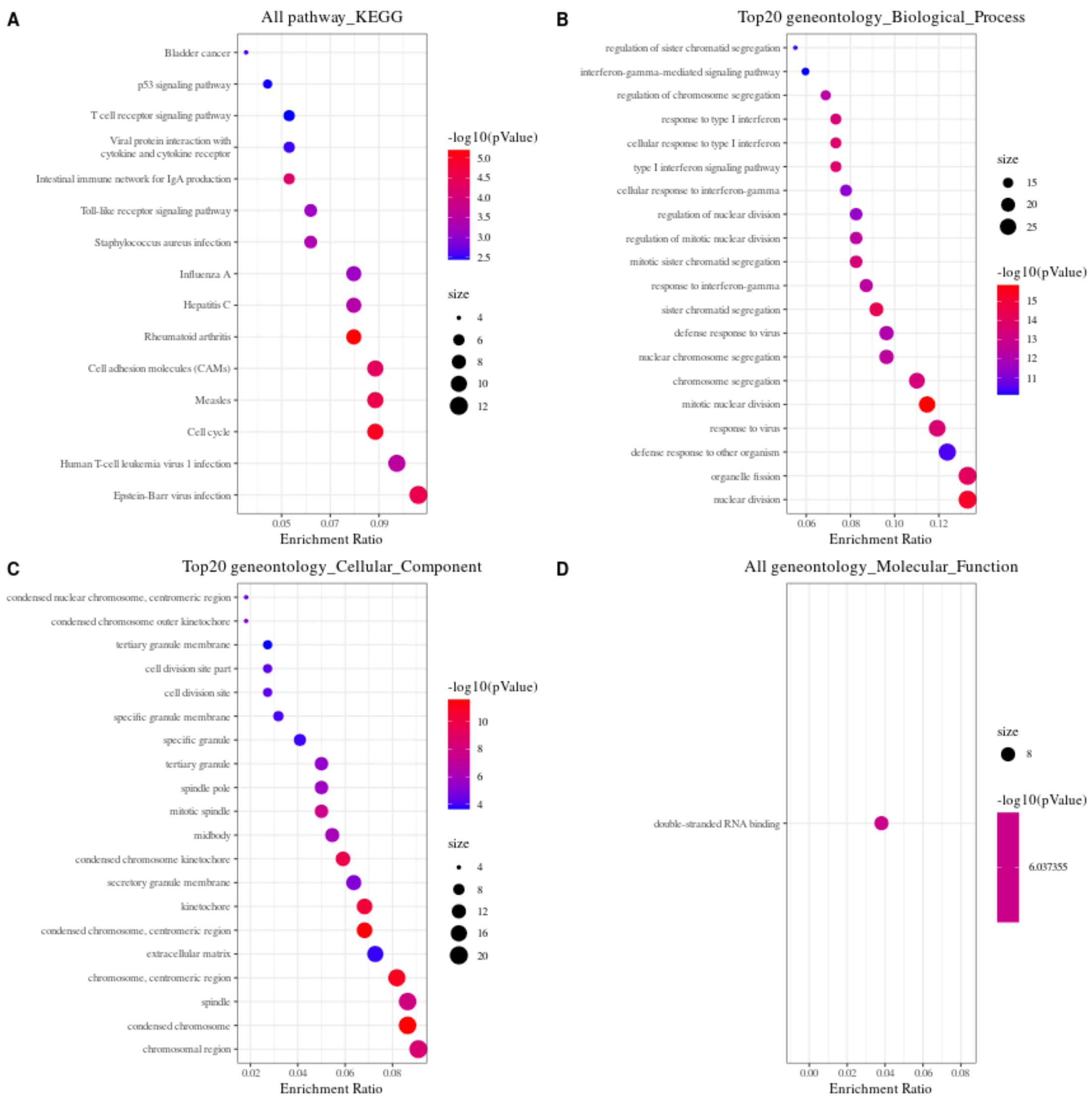

Fig. S 3 KEGG/GO enrichment analysis of co-downregulated genes: (A) KEGG pathway analysis (B) biological process analysis (C) cellular component snslysis (D) molecular funtion analysis

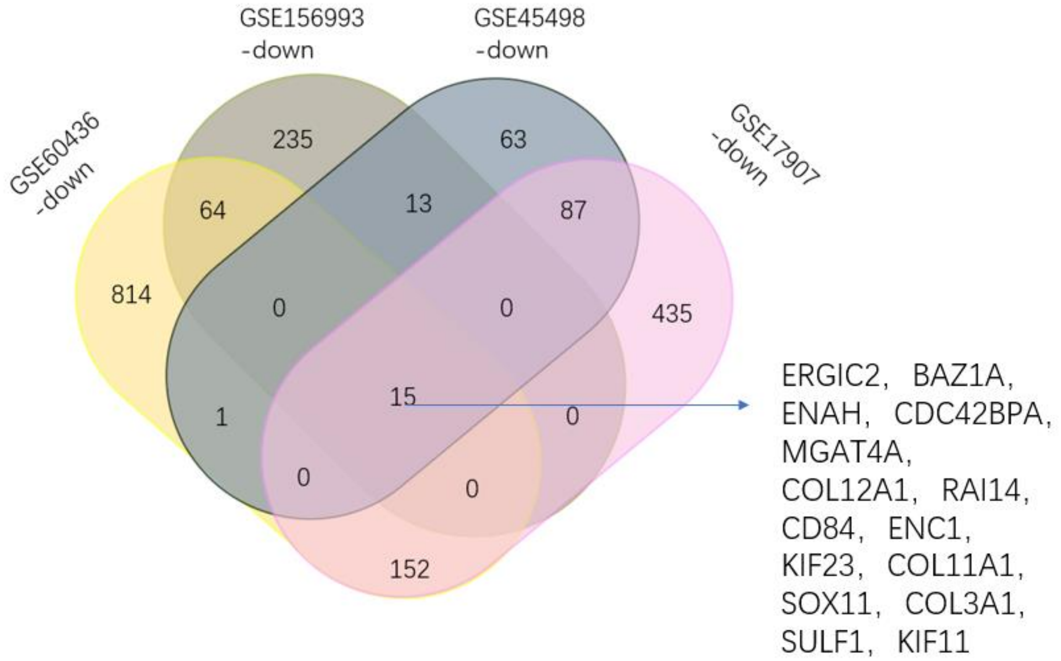

Fig. S 4 Validation analysis of shared genes

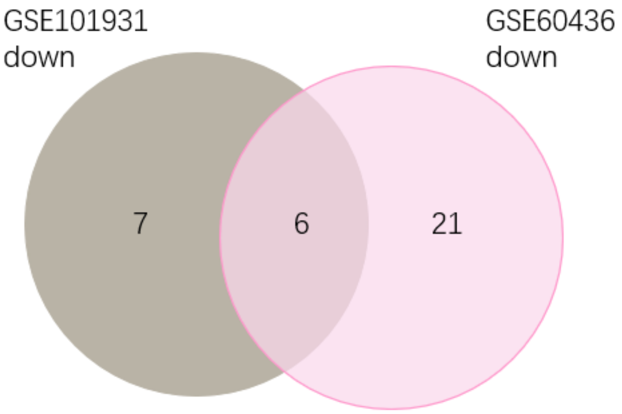

Fig. S 5 Validation analysis of shared miRNAs

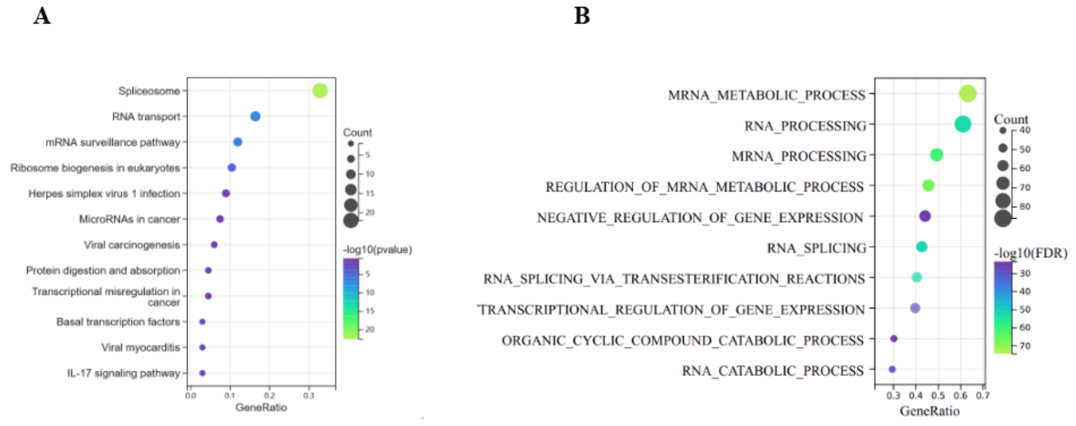

Fig. S 6 BP (biological process) /KEGG enrichment analysis of RBPs

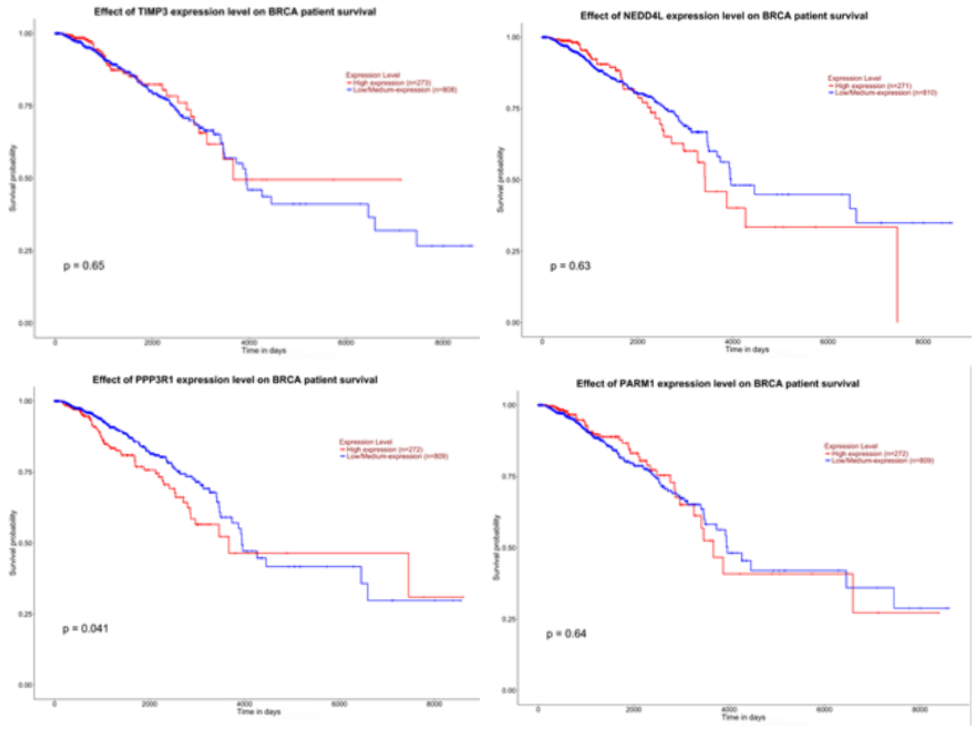

Fig. S 7 survival analysis of other upregulated genes

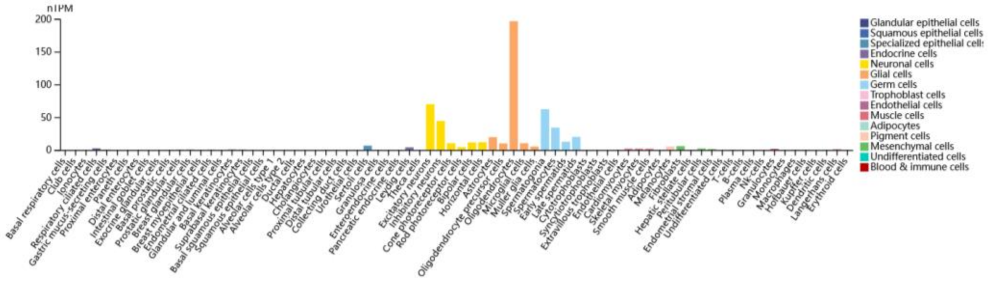

Fig. S 8 Analysis of CPEB1 expression in single cell

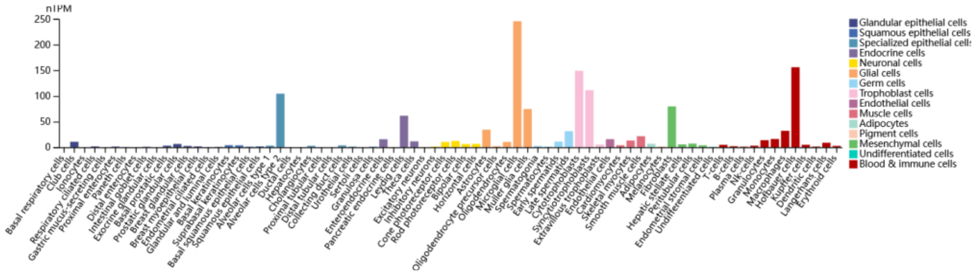

Fig. S 9 Analysis of COLEC12 expression in single cell

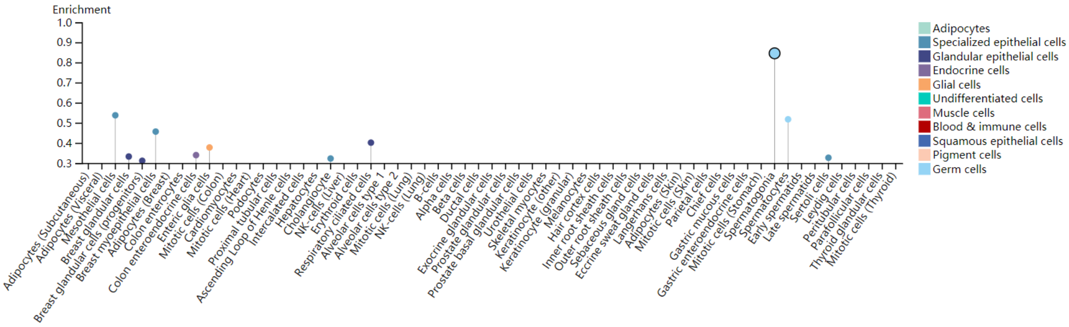

Fig. S 9 Analysis of CPEB1 expression in various tissues

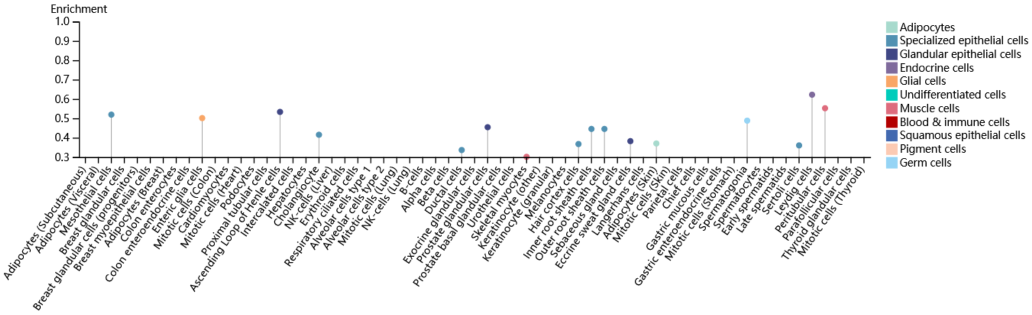

Fig. S 11 Analysis of COLEC12 expression in various tissues
